# Ultrastructural destruction of neurovascular unit in experimental cervical spondylotic myelopathy

**DOI:** 10.3389/fnins.2022.1031180

**Published:** 2022-11-16

**Authors:** Guang-Sheng Li, Xu-Xiang Wang, Ron-Bang Tan, Kang-Heng Wang, Xiao-song Hu, Yong Hu

**Affiliations:** ^1^Spinal Division of Orthopaedic and Traumatology Center, The Affiliated Hospital of Guangdong Medical University, Zhanjiang, China; ^2^Department of Orthopaedics and Traumatology, The University of Hong Kong-Shenzhen Hospital, Shenzhen, China; ^3^Department of Orthopaedics and Traumatology, The University of Hong Kong, Hong Kong, Hong Kong SAR, China

**Keywords:** ultrastructural pathology, neurovascular unit, chronic, compressive, spinal cord injury, cervical spondylotic myelopathy, ultrastructural evidence

## Abstract

**Background and purpose:**

The pathogenesis of cervical spondylotic myelopathy (CSM) remains unclear. This study aimed to explore the ultrastructural pathology of neurovascular unit (NVU) during natural development of CSM.

**Methods:**

A total of 24 rats were randomly allocated to the control group and the CSM group. Basso–Beattie–Bresnahan (BBB) scoring and somatosensory evoked potentials (SEP) were used as functional assessments. Hematoxylin–eosin (HE), toluidine blue (TB), and Luxol fast blue (LFB) stains were used for general structure observation. Transmission electron microscopy (TEM) was applied for investigating ultrastructural characteristics.

**Results:**

The evident compression caused significant neurological dysfunction, which was confirmed by the decrease in BBB score and SEP amplitude, as well as the prolongation of SEP latency (*P* < 0.05). The histopathological findings verified a significant decrease in the amount of Nissl body and myelin area and an increase in vacuolation compared with the control group (*P* < 0.05). The TEM results revealed ultrastructural destruction of NVU in several forms, including: neuronal degeneration and apoptosis; disruption of axonal cytoskeleton (neurofilaments) and myelin sheath and dystrophy of axonal terminal with dysfunction mitochondria; degenerative oligodendrocyte, astrocyte, and microglial cell inclusions with degenerating axon and dystrophic dendrite; swollen microvascular endothelium and loss of tight junction integrity; corroded basement membrane and collapsed microvascular wall; and proliferated pericyte and perivascular astrocytic endfeet. In the CSM group, reduction was observed in the amount of mitochondria with normal appearance and the number of cristae per mitochondria (*P* < 0.05), while no substantial drop of synaptic vesicle number was seen (*P* > 0.05). Significant narrowing of microvascular lumen size was also observed, accompanied by growth in the vascular wall area, endothelial area, basement membrane thickness, astrocytic endfeet area, and pericyte coverage area (rate) (*P* < 0.05).

**Conclusion:**

Altogether, the findings of this study demonstrated ultrastructural destruction of NVU in an experimental CSM model with dorsal–lateral compression, revealing one of the crucial pathophysiological mechanisms of CSM.

## Introduction

Cervical spondylotic myelopathy (CSM) is the most common cervical spinal cord disorder among the elderly population (Amenta et al., 2014). The chronic compression to the cervical spinal cord ultimately causes structural and functional neurovascular destruction in the forms of ischemia, blood–spinal cord barrier (BSCB) disruption, apoptosis of neuron and oligodendrocyte, and axonal demyelination (Baron and Young, 2007; Kalsi-Ryan et al., 2013). However, the vast variation of clinical symptoms and functional presentation among the population of CSM could not be well explained. A good understanding of pathophysiology in CSM is therefore required.

The neurovascular unit (NVU) is a structural and functional complex that plays a vital role in the coupled interaction between neural activity and microcirculation (Lok et al., 2007; Iadecola, 2017; McConnell et al., 2017; Huang et al., 2021). Numerous studies have demonstrated that NVU destruction is involved in the pathological and pathophysiological mechanism of CNS disorders such as brain trauma (Armstead and Raghupathi, 2011; Lok et al., 2015), cerebral ischemia injury (Cai et al., 2017; Zhao et al., 2020), and degenerative cerebral disorders (Cai et al., 2017; Yu et al., 2020). The intercellular connection and interaction among microvascular endothelial cells, perivascular astrocytes, and pericytes regulate the structural and functional integrity of BSCB (Hawkins and Davis, 2005; Sá-Pereira et al., 2012; McConnell et al., 2017). The intercellular coupling between neuron and neuroglial cells has an essential effect on balancing ionic homeostasis, regulating axoplasmic neurotransmission and synaptic re-uptake, and insulating axons to promote nerve conduction velocity (Lok et al., 2007; Guo and Lo, 2009). Thus, the multicellular and multicomponent NVU constitutes a complexed neural–vascular network that is responsible for not only oxygen and nutrition delivery but also intercellular signaling coupling and maintenance of the microenvironmental homeostasis for neural activity (Sá-Pereira et al., 2012; McConnell et al., 2017; Yang et al., 2020; Zhao et al., 2020). In fact, NVU destruction develops prior to motor neuron degeneration (Miyazaki et al., 2011), and NVU remodeling or repair could improve functional recovery (Lake et al., 2017; Chio et al., 2019; Ye et al., 2021). It suggests that NVU could be an early and effective target of treatment. A recent study reported that NVU destruction following the chronic compressive spinal cord injury may lead to impairment of endothelial cell, defect of tight junction, degeneration of neuron and axon, and swelling of astrocyte endfeet and mitochondria (Xu et al., 2017). However, the ultrastructural evidence of NVU is scarce and far from being able to reveal the underlying pathophysiology of CSM (Xu et al., 2017; Guo et al., 2021).

This study explored the ultrastructural pathology of NVU during the natural development of CSM. Pathological staining [hematoxylin–eosin (HE), toluidine blue (TB), and Luxol fast blue (LFB)] and transmission electron microscopy (TEM) examination were used to comprehensively investigate the (ultra)pathological characteristics of NVU in an experimental rat CSM model. Ultrastructural characteristics and notable changes in different aspects of NVU following chronic dorsal–lateral cervical cord compression at the C5 level will be thoroughly examined.

## Materials and methods

### Experimental materials and animal model establishment

A total of 24 female adult Sprague–Dawley (SD) rats (180–250 g) were divided into the CSM group (*n* = 12, cervical spinal cord compression for 2 months) and control group (*n* = 12). All the animal-handling procedures were in accordance with the Guide for the Care and Use of Laboratory Animals, approved by the local Committee on the Use of Live Animals.

**FIGURE 1:**
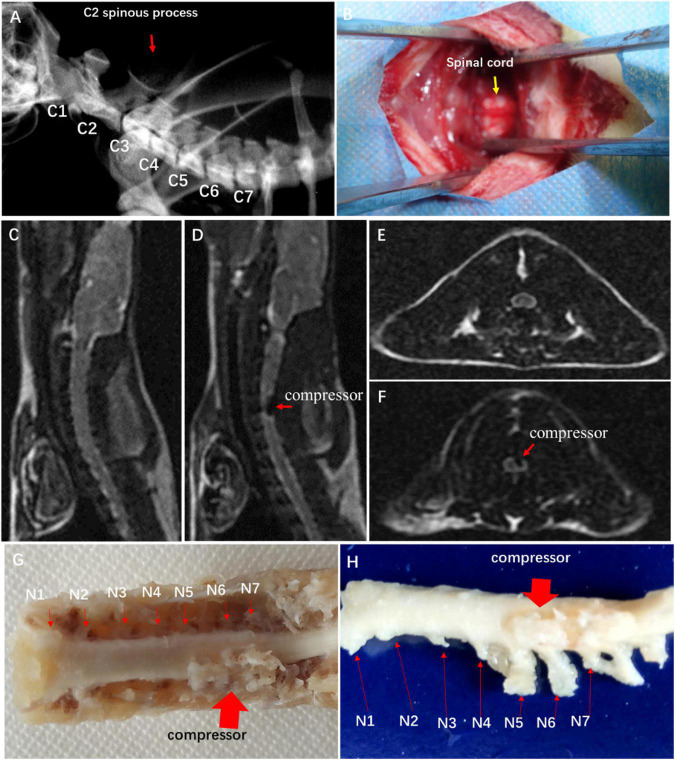
Establishment of experimental CSM rat model. **(A)** Spine location in X-ray fluoroscopy. **(B)** Surgical exposure at C4 and C5 spinal levels. Sagittal MRI T2-weighted images of control group **(C)** and CSM group **(D)**. Axial MRI T2-weighted images of control group **(E)** and CSM group **(F)**. **(G,H)** View of specimen after compression.

In the CSM group, a water-absorbing and progressively expandable synthetic polyurethane polymer sheet (Fulin Ltd., Shenzhen, China) of 3 × 1 × 1 mm was used as an implant material to create compression on the spinal cord (Long et al., 2013). In brief, the rats received general anesthesia with a mixture solution of 10% ketamine and 2% xylazine (Sigma Chemical Co., St. Louis, MO, USA) intraperitoneally. The location of cervical spine levels was identified by X-ray fluoroscopy (Figure 1A). The thin polymer sheet was carefully implanted into the left side of the spinal canal at the C5 level (Figure 1B). The rat after implantation fully recovered from the surgery on a heating bed and was then sent back to the cage freely for food and water. All of the animals in this study survived at the end of observation.

To confirm spinal cord compression, MRI T1- and T2-weighted images were obtained with a 3.0-T MR imager (Discovery MR 750, GE Medical Systems, Milwaukee) 2 weeks after the implantation. Hemorrhage and edema in the epidural space were observed.

### Neurological function assessment

Locomotor function was evaluated by using 21-point Basso–Beattie–Bresnahan (BBB) scoring system in the open field (Basso et al., 1996). The evaluation time point was set at 1 day, 3 days, 7 days, 14 days, 21 days, 1 month, and 2 months postoperatively. Two equally trained spinal surgeons, who were not involved in previous implantation surgeries, were invited to independently evaluate the locomotor function of all rats. The average score was calculated to depict the dynamic change of motor function.

Sensory functional integrity was evaluated by somatosensory evoked potentials (SEP). To elicit cortical SEP, a constant current stimulation with a frequency of 5.1 Hz and a pulse duration of 0.2 ms was applied to the tibial nerve. The cortical SEP was recorded from the skull at location Cz–Fz in the 10–20 system. SEP signals were amplified 100,000 times band-passed between 20 and 2,000 Hz by an electrophysiological measurement system (YRKJ-G2008, Zhuhai, China). The latency and amplitude of the SEP waveform were analyzed after averaging 200 trials.

### Tissues preparation and histopathological examination

All samples from both groups underwent satisfactory euthanasia with sodium pentobarbital solution 2 months postoperatively. Half of the samples from each group were designated to histopathological examination, while the other half reserved for TEM examination.

For histopathological examination, the whole cervical spinal cord was carefully harvested and fixed with 4% phosphate buffer liquid in formaldehyde solution. Then, a 5-mm-long cord block at the C5 level was longitudinally cut and embedded in paraffin. Five-micrometer slides of transverse cord were continuously sectioned for histopathological and immunohistochemical (IHC) staining.

A total count of 12 sectioned specimens were stained with HE (Sigma Chemical Co., St. Louis, MO, USA), TB, and LFB (Sigma Chemical Co., St. Louis, MO, USA). All images of the cords were acquired with a microscope (FV-1000, Olympus, Japan). The number of large motor neurons was counted in the ventral horn of the gray matter at ×10 view for all specimens using ImageJ 1.47v (National Institutes of Health, USA). Nissl body area was examined at ×40 view. After LFB staining, the blue color intensity (×20 view ImageJ) in the posterior funiculus indicated the content of myelin.

### Transmission electron microscopy examination

For TEM examination, the specimen sections were stained with uranyl acetate and alkaline lead citrate for observation with TEM (JEM-1400). Images acquired from the imaging system (GATAN 832) were used to investigate ultrastructural features of neurovascular cytohistology.

Electron micrographs (4,008 × 2,672 pixels, 6.384 × 4.252 μm, 1.6 nm/pixel) of capillaries from the CSM group and control group were compared. Micrographs of microvessels in cross section were taken at 15,000× to measure the area of lumen, endothelial cell, microvascular wall (lumen area was excluded), astrocytic endfeet, and the circumference of microvessel and pericyte with “freehand selections” tool. The pericyte coverage rate (percentage) of microvessel abluminal surface and the total length of the inner pericyte processes around each microvascular abluminal surface were divided by the perimeter of microvessel in 60,000× magnification micrograph. The integrity of tight junction was evaluated by the number of mitochondrial cristae and synaptic vesicle under 100,000× magnification. The tight junctions were recognized as intercellular space when the fluid space is wider than 50 nm.

### Statistical analysis

Sample sizes were determined prior to the experiment. All data are presented as the mean values ± standard error of the mean (SEM). All statistical analyses were performed using SPSS 25.0 software (IBM Corp., Armonk, NY, USA). Comparisons between groups were made using *t*-test of two independent samples. *P* < 0.05 was deemed statistically significant.

## Results

### Image verification of spinal cord compression

Hypointense changes on T1WI and T2WI were observed in the CSM group. The compressor posed dorsal–lateral compression at C5–C6 level and caused evident cord deformation, without spinal cord edema, hemorrhage, and intramedullary cavity (Figures 1C–F). The expanded compressor was encompassed by pseudomembrane inside the spinal canal (Figures 1G,H). A sunken C5 spinal cord was seen on the dorsal–lateral dissected specimen (Figure 1H).

### Neurological dysfunction after chronic cervical cord compression

**FIGURE 2:**
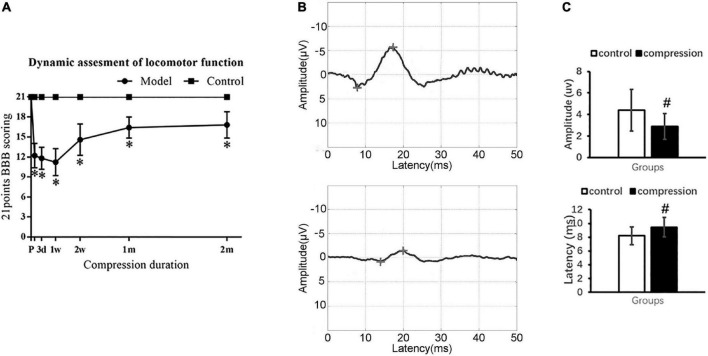
Neurological dysfunction after compression. **(A)** Locomotor evaluation with BBB scores. **(B)** Representative SEP waveforms. **(C)** Values of SEP amplitude and latency in two groups. “*” compare with the control group at the same time point for BBB scores (*P* < 0.05); “#” compared with the control group for SEP (*P* < 0.05); CSM group (*n* = 12), control group (*n* = 12).

The CSM group showed significantly declined BBB scores in contrast to the control group with normal BBB scores at different evaluation time points (*P* < 0.05) (Figure 2A). Ipsilateral upper limb weakness with paw contractures, fore–hind limb discordance, and trunk imbalance were observed in the CSM group. The spontaneous locomotor function recovery was observed 1–2 months postoperatively, before the locomotor function appeared to be in a steady state (16.8 ± 2.0) (Figure 2A).

Somatosensory evoked potential examination confirmed sensory dysfunction in the CSM group (Figure 2B). In the CSM group, there were delayed latency (9.46 ± 1.40 ms at 2 months postoperatively vs. 8.21 ± 1.32 ms preoperatively, *P* < 0.05) and decreased amplitude (2.88 ± 1.21 uv at 2 months postoperatively vs. 4.40 ± 1.93 uv preoperatively, *P* < 0.05) (Figure 2C).

### Motoneuronal and axonal degeneration after chronic compression

**FIGURE 3:**
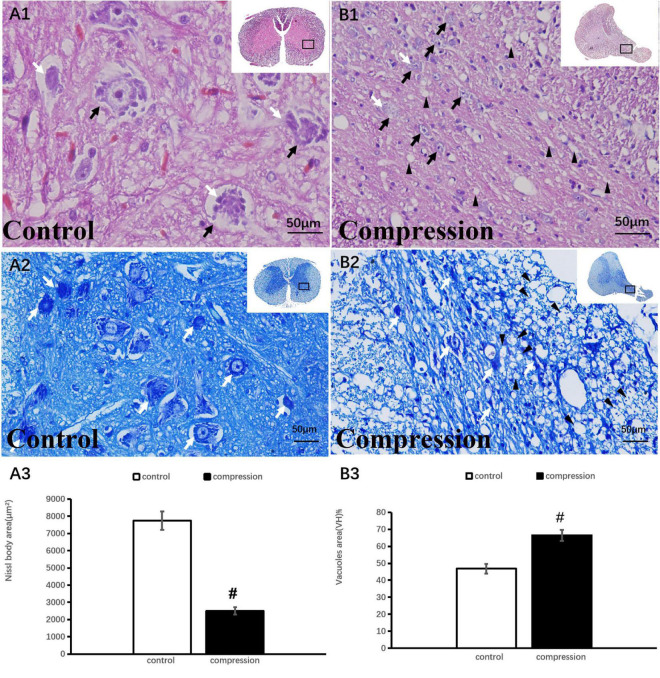
Neuronal degeneration verified by HE and TB staining. **(A1,A2)** Numerous large motor neurons with rich Nissl body were identified in the ventral horn. **(B1,B2)** Loss of neuron and Nissl body with vacuolization in the ventral horn. Black arrow indicates large motor neuron, black arrowhead indicates vacuoles, and white arrow indicates Nissl body. A significant decline in Nissl body **(A3)** and increment in vacuoles **(B3)** in the compression group (*P* < 0.05). “#” significant difference between CSM group and control group (*P* < 0.05, *n* = 6 per group).

In the control group, numerous large motor neurons with a high amount of Nissl body and clear nucleus were identified in the ventral horn (Figures 3A1,A2). In contrast, neuronal death and loss of neuron number were remarkable in the CSM group (Figures 3B1,B2). Disappearance or loss of Nissl body was rather obvious in the large motor neuron (Figures 3B1,B2). Statistical analysis demonstrated a significant decline in Nissl body size (2,753 ± 234 μm^2^ in the CSM group vs. 7,952 ± 543 μm^2^ in the control group, *P* < 0.05) (Figure 3A3). Meanwhile, vacuolization was seen in the ventral horn of the CSM group (Figure 3B1). A significant increment in the vacuole area was found (66.44 ± 3.20 in the CSM group vs. 46.84 ± 2.90 in the control group, *P* < 0.05) (Figure 3B3).

**FIGURE 4:**
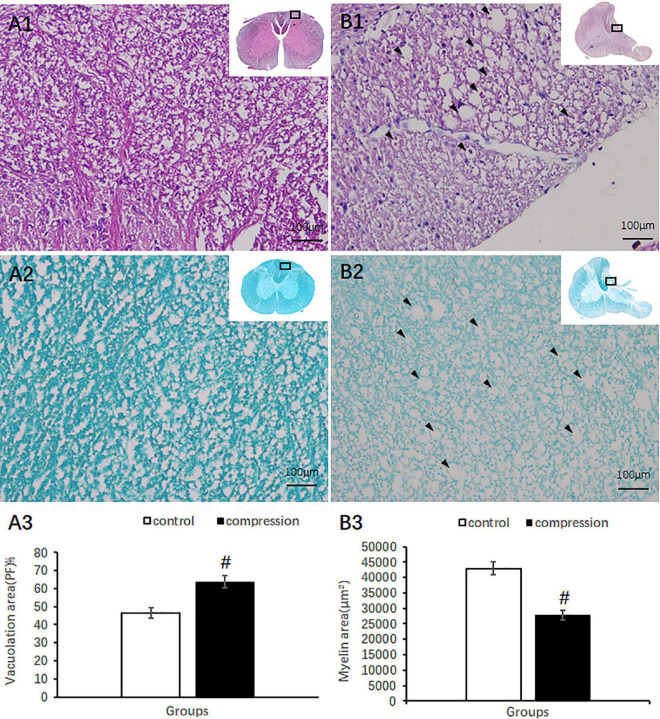
Axonal degeneration in the posterior funiculus after compression revealed by HE and LFB staining. Organized neural fiber derived from the dorsal horn (**A1**, HE staining) and myelin sheath stained in dark blue in the control group (**A2**, LFB staining). **(B1,B2)** Vacuoles formation and a decrease in myelin staining in the posterior funiculus of the CSM group. Vacuolation area increased **(A3)** and myelin area decreased **(B3)** significantly in the CSM group (*P* < 0.05, marked with “#” *n* = 6 per group).

Compared with clear neural fiber derived from the dorsal horn in the control group (Figures 4A1,A2), disrupted neural fiber with increased number of vacuoles was identified in the CSM group (Figures 4B1,B2). A significant increase in the vacuole area around the axon in the posterior funiculus was seen in the CSM group (63.80 ± 3.46) compared with that in the control group (46.64 ± 2.87, *P* < 0.05) (Figure 4A3). LFB staining also showed obvious vacuole formation and a significant decrease in myelin area (27,721 ± 1,587 μm^2^ in the CSM group vs. 42,960 ± 2,153 μm^2^ in the control group, *P* < 0.05) (Figure 4B3).

### Ultrastructural destruction to neurovascular unit after chronic compression

**FIGURE 5:**
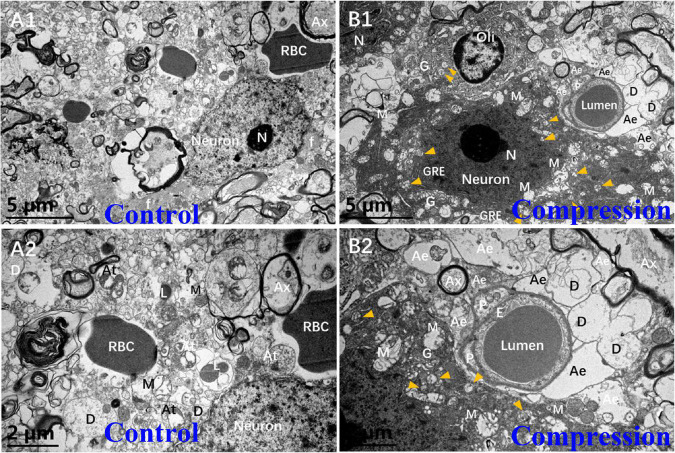
Comparison of ultrastructural evidence of neuron, NVU degeneration, and apoptosis between samples from the control group **(A1,A2)** and CSM group **(B1,B2)**.

In the control group, the Nissl body (Nb), chromatin, and primary lysosome (L) were evenly distributed in karyoplasm (Figures 5A1,A2). The neuropils with several bundles of neurofilaments were clearly distinguished in the gray matter. The mitochondria appeared with clear cristate. In the CSM group, the neurons presented degenerative ultrastructural changes, which were characterized with dense and dark chromatin, plenty of phagolysosomes and autophagic vacuoles, dystrophic neurites, and swelling and disappearance of cristate of mitochondria (Figures 5B1,B2). In particular, increased dense cytoplasm, karyoplasm chromatin condensation and densification, organelles aggregation, and ultrastructural destruction such as nuclear membrane breakdown and nuclei–cytoplasm separation were the most prominent morphological ultrastructural characteristics of apoptotic neuron (Figures 5B1,B2). In addition, a close contact of neuron, oligodendrocyte (Oli), astrocyte endfeet (Ae), and microvessels was seen in the CSM group, which was considered one of the NVU paradigms (Figures 5B1,B2).

**FIGURE 6:**
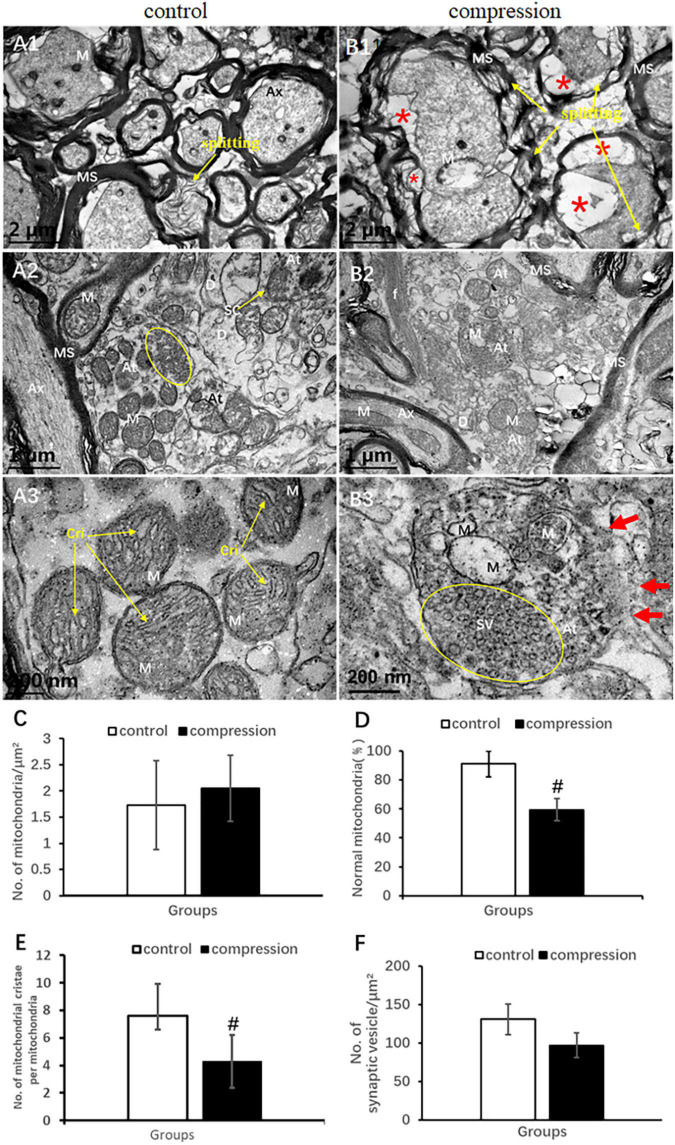
Comparison of ultrastructural destruction of axon and axonal terminal between samples from the control group **(A1–A3)** and CSM group **(B1–B3)**. Increased total number of mitochondria **(C)** but significantly decreased number of mitochondria with normal appearance **(D)** and mitochondrial cristae **(E)** in the CSM group. **(F)** No significant decrease in the number of synaptic vesicles after compression. Ax, axon; MS, myelin sheath; f, filament; At, axonal terminal; SC, synaptic cleft; SV, synaptic vesicle; D, dendrites; M, mitochondria; Cri, mitochondrial cristate; yellow oval include synaptic vesicle “*” axonal cavitation; “#” significant difference between compression and control group (*P* < 0.05, *n* = 6 rat/group).

In the control group, the axons of normal myelinated neural fibers have an even and pale axoplasm with clearly identifiable neurofilaments and microtubules. Some of the mitochondria have distinct cristate (Figure 6A1). The myelinated axons were encompassed with visible lamellae of the myelin sheath. In the CSM group, the swollen axons were found with atrophic axoplasm and cavitation, surrounded by absolutely disorganized and disrupted outer and inner loops of myelin sheaths. Myelin sheath splitting was frequently observed in a large proportion in the neural fibers. Neurofilaments and microtubules were difficult to recognize in the axoplasm in the majority of the neural fibers. Mitochondria were swollen, and mitochondrial cristate disappeared or vacuolized in the CSM group (Figure 6B1).

In the control group, numerous axonal terminals synapsed with dendrite spine and formed mostly asymmetric synapse along with a plenty of spherical synaptic vesicles (Figure 6A2). The axonal terminal axoplasm mainly contained neurofilaments and abundant mitochondria with clear cristate (Figure 6A3). In the CSM group, the axonal terminal appeared to be degenerately changed. Observation included disruption of axonal terminal membrane, disorganized neurofilament, and swelling and vacuolar degeneration of the mitochondrial cristae (Figures 6B2,B3). The proportion of normal mitochondria (59.38 ± 7.76 in the CSM group vs. 91.21 ± 8.94 in the control group) and the number of mitochondrial cristate per mitochondria (4.30 ± 1.93 in the CSM group vs. 7.60 ± 1.72 in the control group) decreased significantly (*P* < 0.05) (Figures 6D,E), whereas no significant difference in the number of synaptic vesicles and total mitochondria (96.97 ± 16.03 in the CSM group vs. 130.84 ± 19.88 in the control group, *P* > 0.05) was found (Figures 6C,F).

**FIGURE 7:**
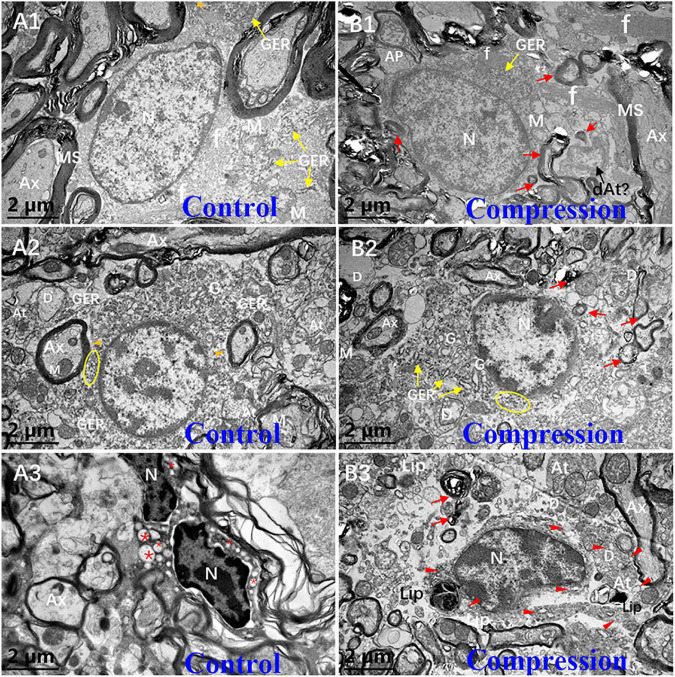
Ultrastructural evidence of neuroglial cells degeneration. Astrocyte **(A1,B1)**, oligodendrocytes **(A2,B2)**, and microglial cells **(A3,B3)** in the control and CSM groups, respectively. N, nucleus; Ax, axon; MS, myelin sheath; f, filament; At, axonal terminal; D, dendrite; GER, granular endoplasmic reticulum; Lip, lipofuscin; L, lysosome; “*” empty phagocytic inclusion; yellow oval includes abundant of free ribosomes; orange arrowhead indicates a few of free glycogen granules in astrocyte; and red arrow indicates degenerating myelin.

In the white matter of the control group, the fibrous astrocytes showed oval nuclei contour with relatively even and lower electron density of karyoplasm and cytoplasm, along with a thin and condense rim of heterochromatin beneath the karyolemma (Figure 7A1). Organelles such as short cisternae of granular endoplasmic reticulum (GER), free glycogen granules, and mitochondria were sparsely distributed in the cytoplasmic matrix, whereas a few of neurofilaments bundles were the most prominent component in the perikaryal cytoplasm. In particular, the astrocyte endfeet (Ae) was seen closely surrounding the microvascular wall (Figures 7B1,B2). In contrast, increased electron density of karyoplasm and cytoplasm was seen in the CSM group (Figure 7B1). The cytoplasm of astrocytes appeared to be filled with degenerating axon (Figure 7B1). The chromatin of oligodendrocytes is clumped and circulating along the karyolemma or scattering throughout the karyoplasm (Figure 7A2). The cytoplasm contains numerous short GER, abundant free ribosomes, well-developed Golgi apparatus (G), and relatively smaller mitochondria. Tight connections between the cytomembrane of oligodendrocytes and some myelinating axons were found in the control group (Figure 7A2). After compression, dissolved karyolemma, degenerating axon, and myelin sheaths inclusions were clearly observed (Figure 7B2).

The microglial cells in the resting state from the CSM group appeared to have elongated nuclei outline, higher density of clumped chromatin, and slightly denser cytoplasm compared with oligodendrocytes in the control group (Figure 7A3). Also, the perikaryal cytoplasm from the CSM group was distended by phagocytosed material, some of which appeared to degenerate myelin and dystrophic dendrite (Figure 7B3). Lipofuscins and autophagy cavitation were seen in the perikaryon (Figure 7B3).

**FIGURE 8:**
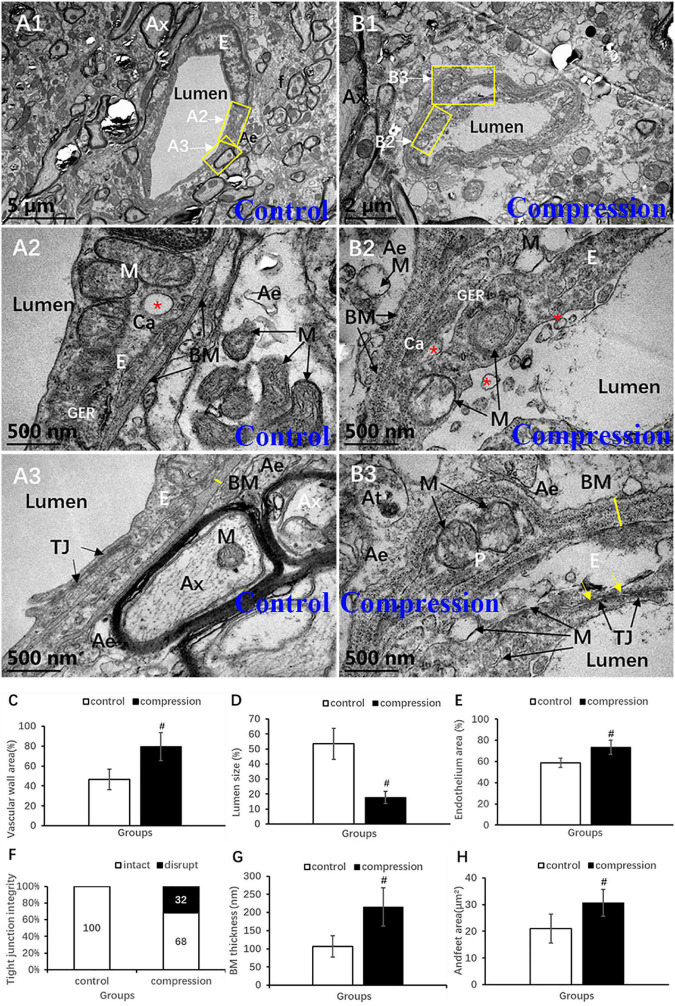
Ultrastructural destruction of microvascular and vascular elements. **(A1–A3)** Normal ultrastructure of microvascular and vascular elements in the control group. **(B1–B3)** Ultrastructural destruction of microvascular and vascular elements in the CSM group. Comparison of characteristics between two groups: vascular wall area **(C)**, endothelium area **(E)**, Tj disruption **(F)**, BM thickness **(G)**, endfeet area **(H)**, and lumen size **(D)**. Ax, axon; E, endothelial cells; p, pericyte; BM, basal membrane; Tj, tight junction; M, mitochondria; Ae, astrocyte endfeet; f, filament; “*” Ca, caveolae; yellow arrow indicates disruption of interacting plasm membranes of Tj; “#” significant difference between CSM and control group (*P* < 0.05, *n* = 6 per group).

Destruction of the vascular elements and collapse of vascular contour were evident in the CSM group (Figures 8A1,B1). Compared with the control group, significant increases were found in the vascular wall area (79.34 ± 14.14 vs. 46.57 ± 10.43, *P* < 0.05) (Figure 8C), endothelium area (73.45 ± 6.58 vs. 58.83 ± 4.35, *P* < 0.05) (Figure 8E), and BM thickness (215.30 ± 52.32 vs. 106.70 ± 29.54, *P* < 0.05) (Figure 8G), while a significant decrease was found in lumen size (17.72 ± 4.14 vs. 53.43 ± 10.43, *P* < 0.05) (Figure 8D). In the control group, the endothelium has distinct and integrated nuclei, karyoplasm, and organelles (Figures 8A1,A2), which was obviously different from the fuzzy and loose appearance of endothelium in the CSM group (Figures 8B1–B3). Loss of electron density of cytoplasmic matrix, mitochondria swelling, disappearing mitochondria, and vacuolation were the main observations of endothelium in the CSM group (Figures 8B2,B3). Such swelling appearance was further proved by the increased area of endothelium in the vascular wall under a chronic compression condition (Figure 8E). After compression, disruption of the interacting plasm membranes of tight junctions and caveolae-like enlargement of the intercellular space were clearly observed (Figures 8B3,F). In the control group, the BM’s electron density was even lower (Figures 8A2,A3). The contour’s corrugated deformation was consistent with the distorted and collapsed vascular profile. Compared with the control group, the thicker BM in the CSM group was scattered with an increased density of electron granules that appeared to be corroding change (Figures 8B3,G).

**FIGURE 9:**
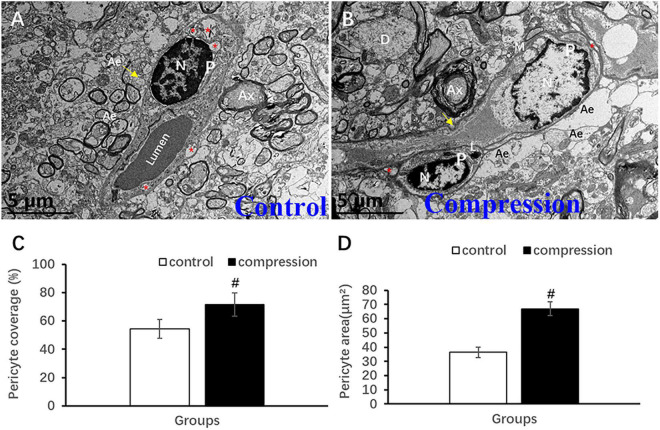
Ultrastructural evidence of pericytes degeneration. **(A)** Control group. **(B)** CSM group. Comparison of pericyte vascular coverage rate **(C)** and pericytes area **(D)**. N, nucleus; P, pericyte; Ax, axon; Ae, astrocyte endfeet; D, dendrite; “*” indicates caveolae. ^#^*P* < 0.05 compared with the control group, *n* = 6 per group.

Unlike the flattened and elongated nuclei of endothelium, pericytes have more roundish or oval nuclei contour (Figures 9A,B). Similar to the oligodendrocyte, the chromatin is quite clumped adjacent to karyolemma and throughout karyoplasm (Figure 9A). In contrast, loss of chromatin was observed in some of the pericytes in the CSM group (Figure 9B). Long and narrow cytoplasmic processes of pericytes crawled and circumvoluted affixed the long axis of microvessels, constituting another physical barrier that strengthened the vascular wall (Figure 9B). The outmost barrier, referred to as astrocytic endfeet, was seen closely affixed to the pericyte or BM. Pericyte vascular coverage rate (%) and pericytes area (μm^2^) were defined as the percentage of ensheathed pericyte length and the area attached to the vascular wall’s long axis, respectively. A significant increment can be seen in the pericyte vascular coverage rate (%) (67.02 ± 4.83 in the CSM group vs. 36.35 ± 3.56 in the control group, *P* < 0.05) and pericytes area (71.48 ± 8.34 in the CSM group vs. 54.41 ± 6.62 in the control group, *P* < 0.05) (Figures 9C,D).

Astrocytic endfeet appeared to be irregularly morphed in cross section with lower electron density (Figures 8A2,B2). In the CSM group, mitochondria expanded and cristae disappeared in most of the perivascular astrocytic endfeet (Figure 8B2), accompanied by a significant increase in perivascular astrocytic endfeet area (30.62 ± 5.03 in the CSM group vs. 21.04 ± 5.40 in the control group, *P* < 0.05) (Figure 8H).

## Discussion

This study comprehensively disclosed the ultrastructural characteristics of NVU and its components’ critical changes after chronic dorsal–lateral compressive spinal cord injury. Ultrastructural observation revealed a series of pathological NVU changes in addition to decreased BBB score, prolonged SEP latency, and reduced SEP amplitude. The NVU destruction appeared with the following ultrastructural characteristics: neuronal degeneration and apoptosis; disruption of axonal cytoskeleton and myelin sheath with dystrophy of axonal terminal; degenerative oligodendrocyte, astrocyte, and microglial cell inclusion with degenerating axon and dystrophic dendrite; swollen microvascular endothelium and loss of tight junction integrity; corroded basement membrane and collapsed microvascular wall; proliferated pericyte and perivascular astrocytic endfeet; and swollen mitochondria in neuron, axon (axonal terminal), and astrocyte. The results of this study have explicitly and systematically demonstrated the ultrastructural destruction of each NVU component in the experimental rat CSM model, which may provide a profound understanding of pathophysiology of CSM. It would help build a research platform for investigating the neurovascular mechanisms behind NVU and finding potential treatment targets to promote an effective therapeutic strategy for CSM.

The surgery-induced compression to the cervical cord at the C5 spinal level was verified at 2 weeks postoperatively using MRI. Neurological dysfunction indicated by changes in BBB score and SEP measurement further validated the establishment of the CSM model as previously reported (Long et al., 2013). It was noted that rats could develop spontaneous functional recovery in 2 months after compressive spinal cord injury, which is different from human. However, behavior, electrophysiology, and pathological findings are useful in simulation of CSM.

In this study, axonal degeneration and demyelination in the CSM group were demonstrated by HE and LFB staining in the posterior funiculus. The ultra-pathological results further verified the loss of Nissl body and large motor neuron in general observation of histopathological findings. The apoptotic changes in large motor neuron in ventral horns during early and mediate phase included increased density of cytoplasm, condensation and densification of karyoplasm chromatin, swelling mitochondria and disappearance of its cristate, breakdown of nuclear membrane and separation of nuclei-cytoplasm, as well as plenty of phagolysosomes and autophagic vacuoles (Figures 5B1,B2). These changes proved that neurons were undergoing apoptosis due to chronic and persistent compression, which is one of the typical neuropathological impairments responsible for neurological dysfunction in CSM (Baptiste and Fehlings, 2006; Yu et al., 2009; Kalsi-Ryan et al., 2013; Karadimas et al., 2013, 2015; Akter et al., 2020). Although ischemia injury and inflammatory impairment have been proposed as the main pathophysiological factors for neuronal apoptosis (Kalsi-Ryan et al., 2013; Karadimas et al., 2015), the underlying mechanisms are still not entirely clear. It is worth noting that the decrease in the number of mitochondria and mitochondrial cristate was the most important characteristic in the degenerative or apoptotic large motor neuron. Thus, more investigations could be focused on the mitochondria-induced pathway in neuronal apoptosis to further elucidate the pathophysiological mechanism of CSM.

Disruption of axolemma, disorganized neurofilament (Figures 6B2,B3), and decreased number of normal mitochondria and mitochondrial cristate in the axonal terminal provided additional particulars for axonal degeneration. The ultrastructural findings were consistent with the axonal degenerative changes revealed by histopathological staining, including increased vacuolation area and decreased myelin area. It may provide an explanation for the underlying pathomechanism of sensory dysfunction, i.e., abnormal SEP latency and amplitude. The axonal terminal (synapse) carrying the neurotransmitter by synaptic vesicle enables interneuron information transmission (Harris and Littleton, 2015). Meanwhile, such context may also have a great impact on mitochondrial viability/renewal and thereby energy supply for synaptic plasticity, axoplasmic transportation, and neurotransmission (Todorova and Blokland, 2017). In brief, these findings may provide ultrastructural evidence for further interpreting the early consensus that long-lasting static and repetitive compression accumulatively produces stretch-associating injury to the axon cytoskeleton (Shi and Pryor, 2002; Baptiste and Fehlings, 2006), disturb axoplasmic transportation, and thereafter cause axonal degeneration, axonal terminal degeneration, and synaptic dysfunction in CSM (Zhou et al., 2015).

In the present study, increased perivascular astrocytic endfeet area was thought to be an astrocytic reaction to chronic compressive injury. Astrocytes can help maintain ion balance around neurons, constitute the BSCB by perivascular astrocytic endfeet, regulate microcirculation (Marina et al., 2020), and mediate transsynaptic signaling in physiological conditions (McConnell et al., 2017). It allows intercellular exchange of other small neutral molecules through aquaporins (Hladky and Barrand, 2016). But the reactive astrocytic is regarded as a detrimental factor that prevents axonal sprouting and plasticity (Okada et al., 2018). It is interesting to note that the astrocytes have numerous inclusions in their cytoplasm, which appeared to be degenerating axon with myelin sheath and axonal terminal (Figure 7B1). Thus, it could be speculated that astrocyte might expedite axonal degeneration or participate in clearing the degenerative axon in chronic compressive circumstances.

In contrast to astrocyte, the oligodendrocytes can be identified by a high density of clumped chromatin, rich and denser cytoplasm, and rich organelles (Figure 7A2). In the gray matter, the oligodendrocyte was frequently seen surrounding the neuron (Figure 5B1), which was called satellite oligodendrocytes, and it also aided in maintaining the normal function of neuron. But in the white matter, the oligodendrocytes defined as interfascicular oligodendrocytes were arranged in rows alongside the axon, providing glucose for neuronal axons under poor nutrition conditions (Hamanaka et al., 2018). The apoptotic changes, which were rather distinct in some of the oligodendrocytes, included strikingly dissolved/disrupted karyolemma, degenerating axon, and myelin sheaths inclusions (Figure 7B2). They indicate that chronic compression may induce the apoptosis of oligodendrocyte. Our findings further support the previous conclusions that apoptosis of oligodendrocyte may lead to axonal demyelination, neuronal impairment, and neurological dysfunction in CSM (Kim et al., 2003; Baptiste and Fehlings, 2006; Yu et al., 2009, 2011; Karadimas et al., 2010, 2013; Kalsi-Ryan et al., 2013).

In the CSM group, we also found that perikaryal cytoplasm was distended by phagocytosed material, some of which appeared to be degenerating myelin, dystrophic dendrite, lipofuscins, and autophagy cavitation (Figure 7B3). The findings indicate that microglial cells were involved in promoting phagocytosis and clearing degenerated neural tissues. Hence, microglial cells often serve as “daring vanguard” or “maintainer” who could detect any subtle damage in microenvironment and clear the degenerated tissues and injury debris (Liu L. R. et al., 2020). Moreover, neuroinflammatory impairments regulated by microglial cell are one of the key pathophysiological causes that lead to neuronal and neuroglial cell death (Glass et al., 2010; Savage et al., 2018). In immunological and inflammatory environment, microglial cells may develop into M1-like phenotype (classical activated macrophages) or M2-like phenotype (alternative activated macrophages) (Orihuela et al., 2016). M1-like phenotype is associated with secretion of inflammatory cytokines such as tumor necrosis factor alpha (TNF-α), interleukin-1 (IL-1), and interleukin-6 (IL-6) and is regarded as a detrimental phenotype. Meanwhile, M2-like phenotype is able to promote anti-inflammatory cytokine expression such as interleukin-10 (IL-10), transforming growth factor beta (TGF-β), and glucocorticoids and is regarded as a beneficial phenotype (Liu L. R. et al., 2020). In an experimental rat CSM model, M1-like phenotype microglial cell existed chronically and may be responsible for neuronal and axonal degeneration, while M2-like phenotype appeared only temporally (Hirai et al., 2013). Thus, inducing the development of M2-like phenotype while inhibiting M1-like phenotype by changing the cellular microenvironment to prevent inflammatory impairment seems to be a promising therapeutic strategy for CSM (Plastira et al., 2019).

Blood–spinal cord barrier is one of the key components of NVU that is comprised of, from inside to outside, tightly connected endothelial cells, the encapsulated basement membrane, the adhesive pericyte, and the enwrapping astrocytic endfeet (Hawkins and Davis, 2005; Choi and Kim, 2008). The interactions and reciprocation among those components construct the functional integrity of BSCB (Choi and Kim, 2008). BSCB disruption is one of the key pathophysiological processes in the CSM (Karadimas et al., 2013; Blume et al., 2020), and the magnitude of BSCB disruption was strongly correlated with the severity of myelopathy (Blume et al., 2020). In this study, microvascular ultrastructural destruction induced by chronic compression was characterized by swollen endothelial cell, expanded and corrugated basement membrane, and collapsed microvascular contour and luminal stenosis (Figures 8B2,B3). The findings were consistent with the previous ultrastructural features depicted (Xu et al., 2017). However, this study presented the swollen mitochondria with disrupted mitochondrial cristae in the endothelial cells as a potential pathomechanism responsible for endothelial dysfunction. We also observed disrupted integrity of tight junctions in the forms of disruption of interacting plasm membranes and caveolae-like enlargement of intercellular space (Figure 8B3). Such disrupted integrity leads to the increase in BSCB permeability. Tight junctions are the main inter-endothelium connections that strengthen the physical barrier that controls paracellular substance diffusion through the vascular wall into the parenchyma of spinal cord. The tightly connected endothelial cells and tight junction gateways enable physiological transcellular flux that is responsible for nutrition delivery from peripheral blood and metabolism substance transfer from the neural tissues (Bartanusz et al., 2011; Daneman and Prat, 2015).

Our findings verified that chronic compression led to the corrosion and expansion of basement membrane (Figure 8B3). In addition, the abnormally thickened basement membrane was arranged in a loose pattern and had lower electron density and indistinct lamellar structure (Figure 8B3). This indicates the swollen expansion rather than the proliferation of basement membrane in chronic compressive circumstances. The basement membrane, mainly composed of laminin and collagen IV (Hallmann et al., 2005), encapsulates the microvascular endothelial cell. Besides, deformation contour of basement membrane was also thought to contribute to the collapse of microvascular wall. Accordingly, it can be inferred that the collapse of microvascular wall and stenosis of vascular lumen may reduce spinal cord perfusion. It is known that extracellular matrix enzymes such as matrix metalloproteases (MMP) are essential in regulating the metabolic balance of basement membrane and extracellular matrix. However, an intemperate expression of MMP-9 may speed up degradation process of basement membrane and lead to NVU destruction (del Zoppo, 2010).

Pericytes are embedded in the basement membrane, and they wrapped around the abluminal surface of microvessel, maintaining structural and functional integrity of microvessel (Brown et al., 2019; Liu Q. et al., 2020). In the present study, the cytoplasmic processes of pericytes were observed to crawl and circumvolute affixed the long axis of microvessel (Figure 9B), constituting another physical barrier for the vascular wall. In the CSM group, loss of chromatin may indicate degeneration in some of the pericytes (Figure 9B) and could be associated with increased microvascular permeability (Armulik et al., 2010). A recent study also demonstrated that pericyte degeneration reduced microvascular blood flow and oxygen supply, leading to NVU dysfunction and neurodegeneration (Kisler et al., 2017). We also found that pericyte vascular coverage rate and pericytes area increased significantly in the CSM group (Figures 9C,D). Similar findings from the previous study showed that the coverage rates of pericytes along the long axis of vascular wall were 54 and 71% in the control and CSM groups, respectively, exceeding the 22–32% coverage rate of cerebral capillary surface (Fisher, 2009). Meanwhile, it remains unanswered whether such a difference was a compensatory proliferating response to chronic compression. In addition, caveolae-like vesicle/vacuoles were also commonly seen in the cytoplasm of pericytes as in the endothelium in both groups (Figures 9A,B), suggesting that pericyte may participate in transcellular exchange between the blood and the spinal cord parenchyma (Xu et al., 2017). Besides, pericyte could promote development of endothelial cells (Hellström et al., 2001), maturation of microvasculature (Fisher, 2009), and repairment of destructed NVU components potentially on behalf of the stem cell (Fisher, 2009). In brief, pericyte plays an important role in the regulation of microvessel and microcirculation homeostasis (Sá-Pereira et al., 2012).

Compared with the progressive neurological deterioration of CSM in human, rats usually show spontaneous functional recovery in 2 months, disallowing a long-term study over 2 months. Clinical trials are needed for a translation study (Nishida et al., 2012). The present study applied dorsal–lateral compression to the spinal cord as animal CSM model. It is a question of whether the NVU changes would vary after ventral compression was applied. Further development of animal models with ventral compression is needed to observe variation of NVU changes in different compression types.

## Conclusion

In summary, we have established explicit and systematic ultrastructural evidence of NVU destruction in the present experimental CSM model with dorsal–lateral compression. The ultrastructural changes have the following characteristics: (1) neuronal degeneration and apoptosis; (2) disruption of axonal cytoskeleton (neurofilaments and microtubules) and myelin sheath with dysfunction mitochondria; (3) disruption of axolemma and dystrophy of axonal terminal with dysfunction mitochondria; (4) degenerative oligodendrocyte, astrocyte, and microglial cell inclusion with degenerating axon and dystrophic dendrite; (5) swollen endothelium with dysfunction mitochondria and loss of tight junction integrity; (6) expanded and corrosive change of basement membrane with collapsed contour of microvascular wall; (7) increment in pericyte vascular coverage rate (area); and (8) increased perivascular astrocytic endfeet area with significant dysfunction mitochondria. Above all, neuronal and axonal degeneration and ultrastructural destruction of cellular constituents and organelles, such as dysfunction of mitochondria, were most evident in the present study. Microvascular collapse and compensatory changes in the forms of expanded basement membrane and proliferated pericytes and astrocytic endfeet were remarkable. These characteristics may inspire further pathophysiological investigation on the potential target NVU component of treatment to promote an effective therapeutic strategy.

## Data availability statement

The original contributions presented in this study are included in the article/supplementary material. Further inquiries can be directed to the corresponding author.

## Ethics statement

This animal study was reviewed and approved by the Committee on the Ethics of Animal Experiments of the Affiliated Hospital of Guangdong Medical University.

## Author contributions

G-SL and YH were involved in the study design and drafted the manuscript. G-SL, X-XW, K-HW, and X-SH were involved in the experimental implementation. G-SL and R-BT were involved in the data acquisition. G-SL analyzed the data. YH reviewed the manuscript. All authors read and approved the final version of the manuscript.
